# Management of pilonidal sinus by *Kshar Sutra*, a minimally invasive treatment

**DOI:** 10.4103/0974-7788.64408

**Published:** 2010

**Authors:** Amar P. Dwivedi

**Affiliations:** *Department of Shalya Tantra, Dr. D.Y. Patil Ayurved College and Hospital, Nerul, Navi Mumbai, India*

**Keywords:** Pilonidal sinus, *Shalya Tantra*, *Nadi Vrana chikitsa*, *Kshar Sutra*

## Abstract

A Pilonidal sinus (PNS) occurs in the cleavage between the buttocks (natal cleft) and can cause discomfort, embarrassment and absence from work. It is more common in men (as they have more hair) than in women. The most commonly used surgical techniques for this disorder include excision and primary closure and excision with reconstructive flap. However, the risk of recurrence or of developing an infection of the wound after the operation is high. Also, the patient requires longer hospitalization, and the procedure is expensive. There is a similarity between *Shalyaj Nadi Vran* described in *Sushruta Samhita* and Pilonidal sinus. Sushruta has advocated a minimally invasive para-surgical treatment, *viz., Kshar Sutra* procedure, for *nadi vran*. Hence this therapy was tried in Pilonidal sinus, and is described in this case report. *Kshar Sutra* treatment not only minimizes complications and recurrence but also enables the patient to resume work quicker and with less discomfort, impact upon body image and self-esteem as well as reduced cost.

## INTRODUCTION

A Pilonidal sinus is a sinus track which commonly contains hair. It occurs under the skin between the buttocks (the natal cleft) at a short distance above the anus. The sinus track goes in a vertical direction between the buttocks. Most cases occur in young male adults. The origin of Pilonidal disease is not fully understood, although hormonal imbalance, presence of hair, friction and infection are often implicated.[1]

The most commonly used therapy is surgery including wide excision and healing by secondary intention. However, post operative recurrence following surgery is high, leading to frequent and time-consuming wound care. Hence, there is a need to evaluate the role of the other alternative/ innovative techniques for the management of this challenging disease so as to minimise recurrence , make it cost effective, with improved acceptability & minimum hospitalization.

The 'Sushrut Samhita',[2] describes a condition *'Shalyaj Nadi Vran'* which is similar to 'Pilonidal sinus'. *'Shalyaj nadi vran'* is a track which is described to be due to presence of pus, fibrosed unhealthy tissue & hair etc. inside left unnoticed. Sushruta has advocated a very unique minimally invasive treatment i.e. '*Kshar Sutra*' procedure for management of *Nadi vran* (PNS).

## CASE REPORT

A 33 year old male patient aged, came to the Surgery O.P.D at Dr. D.Y. Patil Ayurvedic Hospital, Nerul, Navi Mumbai with complaints of recurrent discharge from a boil over an operated site along with pain and discomfort in October 2008.

He gave a history of Z-plasty performed for Pilonidal sinus performed in 2005. The disease re-occurred after 3 years in 2008 and this was confirmed by CT Scan. The patient was not willing for surgery again and requested Ayurvedic treatment. Hence, *Kshar Sutra* procedure was offered.

Before planning treatment other etiologies like Tuberculosis, Pelvic inflammation causing abscess, HIV, diabetes mellitus, foreign body or trauma were ruled out.

**Figure 1:**
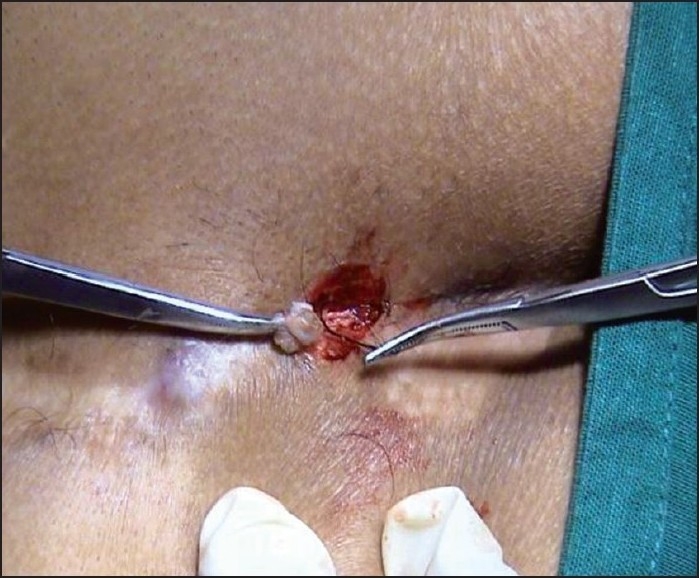
External openings coinciding with natal cleft excised under LA (embedded hair follicles were removed).

**Figure 2:**
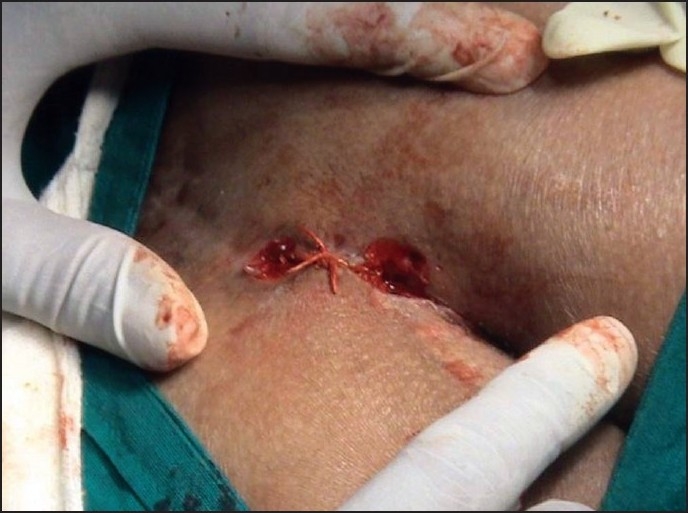
Ligation of *Kshar Sutra* in the underlying track (sinus)

After confirmation of the pilonidal sinus by CT Scan, the two external openings were excised under local anesthesia and the embedded hair follicles were removed [Figure 1]. The *Kshar Sutra* was tied covering the entire underlying track for simultaneous cutting and healing [Figure 2]. Appropriate dressing was given under aseptic conditions. The patient was discharged on the day after the procedure.

**Figure 3:**
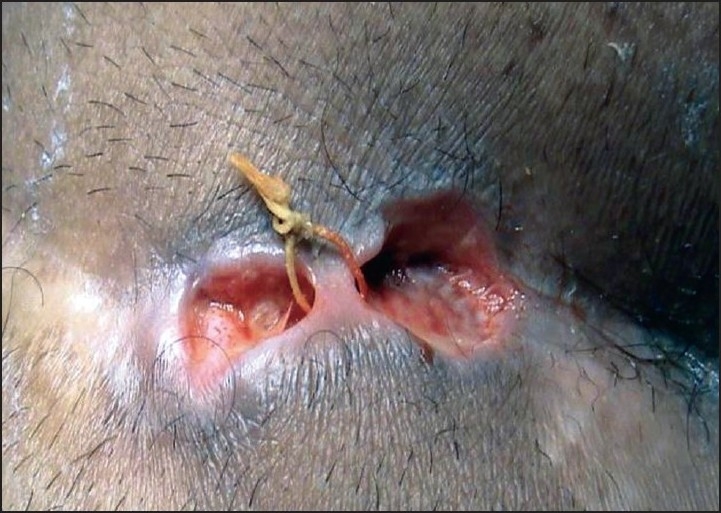
Second changing of *Kshar Sutra* (after 7 days)

Patient was asked to attend surgical clinic for dressing on alternate days. Seitz bath (hip) with lukewarm water was advocated before dressing. The *Kshar Sutra* was changed weekly for 3 sittings [Figure. 3]. To promote healing and reduce pain & inflammation oral antibiotics, anti-inflammatory drugs and multi vitamins were also prescribed.

**Figure 4:**
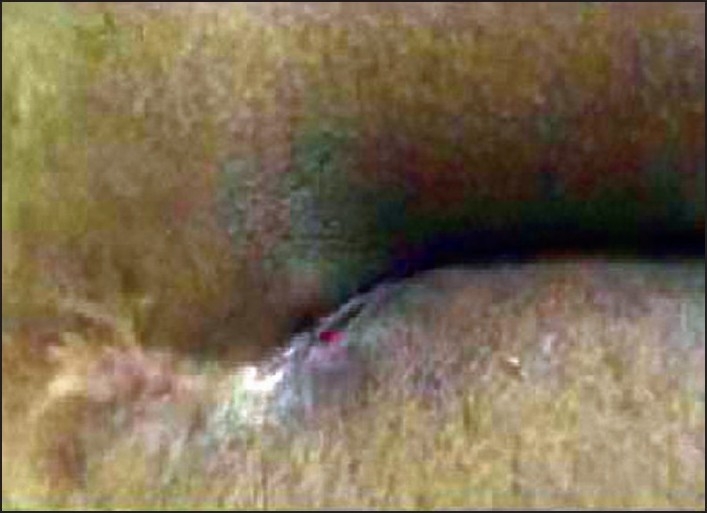
Completely healed track (after 21 days)

The tracks cut through and simultaneously healed by 4 weeks [Figure. 4]. However, it was observed that healing rate was slow compare to cutting rate and the patient was observed for a period of one year to check for recurrence.

*Kshar Sutra* is a medicated thread (seton) coated with herbal Alkaline drugs like Apamarga (*Kshar*) (Ash of *Achyranthus ascera*), Snuhi (*Euphorbia nerufolia*) latex and Haridra (*Curcuma longa*) powder in a specific order. This combination of medicines on the thread helps in debridement and lysis of tissues, exerts antifungal, anti bacterial, and anti inflammatory. Another mechanism proposed for the *Kshara Sutra* is that it destroys the residual glands in the epithelium.

## DISCUSSION

This minimally invasive procedure *Kshar Sutra* has good potential in the management of Pilonidal sinus. It minimizes rates of complication and recurrence and enables the patient to resume work and normal social activities as early as possible. It is an acceptable treatment to the patient in terms of cost of treatment, extent of discomfort, impact upon body image and self-esteem.
